# Effectiveness and safety of levodopa–entacapone–carbidopa infusion in Parkinson disease: A real‐world data study

**DOI:** 10.1111/ene.16535

**Published:** 2024-10-28

**Authors:** Diego Santos‐García, Lydia López‐Manzanares, Inés Muro, Pablo Lorenzo‐Barreto, Elena Casas Peña, Rocío García‐Ramos, Tamara Fernández Valle, Carlos Morata‐Martínez, Raquel Baviera‐Muñoz, Irene Martínez‐Torres, María Álvarez‐Sauco, Déborah Alonso‐Modino, Inés Legarda, María Fuensanta Valero‐García, José Andrés Suárez‐Muñoz, Juan Carlos Martínez‐Castrillo, Ana Belén Perona, Jose María Salom, Esther Cubo, Caridad Valero‐Merino, Nuria López‐Ariztegui, Pilar Sánchez Alonso, Sabela Novo Ponte, Elisa Gamo González, Raquel Martín García, Raúl Espinosa, Mar Carmona, Cici Esmerali Feliz, Pedro García Ruíz, Teresa Muñoz Ruíz, Beatriz Fernández Rodríguez, Marina Mata

**Affiliations:** ^1^ Department of Neurology Hospital Universitario de A Coruña, Complejo Hospitalario Universitario de A Coruña A Coruña Spain; ^2^ Grupo de Investigación en Enfermedad de Parkinson y otros Trastornos del Movimiento Instituto de Investigación Biomédica de A Coruña A Coruña Spain; ^3^ Hospital San Rafael A Coruña Fundación Degen A Coruña Spain; ^4^ Complejo Hospitalario Universitario de A Coruña A Coruña Spain; ^5^ Hospital Universitario la Princesa Madrid Spain; ^6^ Hospital Clínico Universitario San Carlos Madrid Spain; ^7^ Hospital de Cruces Bilbao Spain; ^8^ Hospital Universitario la Fe Valencia Spain; ^9^ Hospital General Universitario de Elche Elche Ethiopia; ^10^ Hospital Universitario de la Candelaria Santa Cruz de Tenerife Spain; ^11^ Hospital Universitario Son Espases Palma de Mallorca Spain; ^12^ Hospital Dr. Negrín Las Palmas de Gran Canaria Spain; ^13^ Hospital Universitario Ramón y Cajal Madrid Spain; ^14^ Complejo Hospitalario Universitario de Albacete Albacete Spain; ^15^ Hospital Clínico Universitario de Valencia Valencia Spain; ^16^ Hospital Universitario de Burgos Burgos Spain; ^17^ Hospital Arnau de Vilanova Valencia Spain; ^18^ Hospital Universitario de Toledo Toledo Spain; ^19^ Hospital Puerta de Hierro Madrid Spain; ^20^ Hospital Universitario de Jerez Cádiz Spain; ^21^ Hospital Universitario de Basurto Bilbao Spain; ^22^ Hospital Fundación Jiménez Díaz Madrid Spain; ^23^ Hospital Regional Universitario de Málaga Malaga Spain; ^24^ Hospital Infanta Sofía Madrid Spain

**Keywords:** effectiveness, infusion, levodopa–entacapone–carbidopa, Parkinson disease, safety

## Abstract

**Background and purpose:**

Levodopa–entacapone–carbidopa intestinal gel (LECIG) infusion is a recently developed device‐aided therapy for advanced Parkinson disease (PD) patients. The aim of this study was to report real‐world evidence about the effectiveness, tolerability, and safety of LECIG in PD patients.

**Methods:**

A multicenter observational retrospective study of the first patients who initiated LECIG in Spain was performed. All neurologists with an experience of at least two patients treated until 30 March 2024 were invited to participate. Data about effectiveness and safety from the medical records (V0, pre‐LECIG; V1, initiation of LECIG; V2, post‐LECIG follow‐up) with a total of 246 variables were collected.

**Results:**

Seventy‐three PD patients (61.6% males, 70.1 ± 9.1 years old) from 21 Spanish centers with a mean disease duration of 14.4 ± 6.3 years (range = 5–31) were included. Twenty‐six patients (35.6%) were switched directly from levodopa–carbidopa intestinal gel. The mean exposure to LECIG was 177.3 ± 110.5 days (range = 7–476). The mean daily OFF time decreased from 5.2 ± 3 (pre‐LECIG) to 1.9 ± 1.8 (post‐LECIG; *n* = 66, *p* < 0.0001). Global improvement was observed in >85% of the patients. No significant change was detected in the levodopa equivalent daily dose from V0 to V2. Only 7% received 24‐h infusion, and 24.7% required more than one cartridge per day at V2. Thirty‐four patients (46.6%) had at least one adverse event related to LECIG and/or the device system. Five patients (6.8%) discontinued LECIG.

**Conclusions:**

LECIG was safe and effective in advanced PD patients.

## INTRODUCTION

Parkinson disease (PD) is the second most frequent neurodegenerative disorder after Alzheimer disease. Although there is no cure, symptoms can improve with symptomatic dopaminergic treatment, with levodopa being the gold standard treatment for PD [1]. During the first steps of PD, the response is stable throughout the day. However, with the progression of the disease, the clinical response to levodopa becomes complicated by a reduction in the duration and reliability of motor improvement (motor fluctuations) and the emergence of involuntary movements (levodopa‐induced dyskinesia) [2]. Recent clinical research provides growing evidence that various nonmotor symptoms (NMSs) such as neuropsychiatric, autonomic, and sensory symptoms (particularly pain) also show fluctuations in patients with motor fluctuations (called nonmotor fluctuations) [3]. Clinical fluctuations (motor and/or nonmotor) are very frequent even in early PD patients [3] and can be treated with a proper optimization of levodopa or by adding different drugs [4]. Moreover, device‐aided therapies (DATs; i.e., deep brain stimulation and infusion therapies) can be indicated in correctly selected patients not properly controlled with conventional treatment [5]. Among them, levodopa–carbidopa intestinal gel (LCIG) treatment has been shown to be safe and effective, with many studies reporting an increase and decrease in ON and OFF time, respectively, with improvement of motor symptoms, NMSs, quality of life, and autonomy for activities of daily living [6]. Furthermore, LCIG has been used in many countries for >20 years [7]. But as a novel DAT option, the triple combination levodopa–entacapone–carbidopa intestinal gel (LECIG; Britannia Pharmaceuticals) has recently been approved in some countries to use in advanced PD patients [8]. LECIG contains the catechol‐O‐methyltransferase (COMT) inhibitor entacapone (20 mg/mL) in addition to levodopa (20 mg/mL), and due to this, the bioavailability of levodopa from LECIG infusion is 20%–25% higher than that from LCIG infusion [9, 10]. Regarding this aspect, a benefit of LECIG over LCIG is a reduced daily dose of levodopa requirement, with the possibility of using a smaller pump system and to be a better therapeutic option in patients at risk of polyneuropathy [11]. However, there are only a few studies published about the effect and safety of LECIG in advanced PD patients [10, 11, 12, 13, 14, 15]. Waiting for the results of the ongoing phase 4 study ELEGANCE [16], real‐world evidence (RWE) is required.

The aim of this study was to report RWE about the effectiveness, tolerability, and safety of LECIG in PD patients treated by movement disorders specialists at different centers from Spain.

## MATERIALS AND METHODS

Patients with advanced PD treated with LECIG were included in this multicenter, longitudinal, retrospective, observational study. Centers from Spain with an experience of at least two PD patients treated with LECIG until 31 March 2024 were invited to participate. All patients treated with LECIG at each center had to be included. The data were collected from three different time points: V0, indication of therapy (LECIG) by the neurologist; V1, initiation of LECIG; and V2, a follow‐up visit. The data of visits V0 and V1 were collected from the medical records, whereas the data of visit V2 were collected from a specific data report registry assessed in clinic. The period for collecting the data was 6 months, from December 2023 to May 2024.

Information on sociodemographic aspects, comorbidity, factors related to PD, and treatment was collected. Both the Hoehn and Yahr (H&Y) stage and the Unified Parkinson's Disease Rating Scale Part III (UPDRS‐III) score during the OFF and ON states were collected at V0 and during the ON state at V2. Data about OFF time (hours per day) and the presence of different disabling motor symptoms and NMSs according to the neurologist opinion was also collected at V0 and V2. The Clinical Global Impression of Change (CGI‐C) scale was applied at V2 to record the impression of the neurologist, principal caregiver, and patient. All information about the LECIG initiation and changes in treatment including the levodopa equivalent daily dose (LEDD) [17] was recorded. Homocysteine and folate, B6, and B12 vitamin levels and weight were measured at V0 and at V2. Adverse events (AEs) and discontinuations of treatment were collected from the follow‐up.

### Statistical analysis

Data were processed using SPSS 20.0 for Windows. Different variables were expressed as quantitative and/or qualitative variables. Distribution for variables was verified by one‐sample Kolmogorov–Smirnov test. The primary efficacy outcome was the change from baseline (V0) to the end of the observational period (V2) in the OFF time. Wilcoxon rank‐sum was applied. The change from V0 to V2 in other variables was assessed using nonparametric tests (Wilcoxon rank‐sum vs. McNemar test vs. marginal homogeneity test depending on the variable type). Values of *p* < 0.05 were considered significant. Safety analyses were assessed by AEs. All AEs were coded using the current version of the Medical Dictionary for Regulatory Activities (MedDRA). The number and percentage of subjects with treatment‐emergent AEs by the MedDRA system organ class and preferred term, by the relationship to study treatment as assessed by the investigator, were provided for overall subjects. A total of 246 variables were collected for the analysis.

### Standard protocol approvals, registrations, and patient consent

For this study, we received approval from the Comité de Ética de Investigación de medicamentos de Galicia of Spain (2023/527; 19 November 2023). Written informed consent from all participants (patients and controls) in this study was obtained.

## RESULTS

A total of 73 PD patients were included in the study (61.6% males, 70.1 ± 9.1 years old). Mean disease duration was 14.4 ± 6.3 years (range = 5–31). At V0, mean OFF time (*n* = 70) was 5.3 ± 2.9 h (range = 1–15), and 72.6% and 83.6% of the patients had nonmotor fluctuations and dyskinesia, respectively. Postural instability gait difficulty was the most frequent motor phenotype (38.9%), and 32.9% of the patients had cognitive impairment (only one had dementia; Table 1). Up to 37% of the patients (*n* = 70) had not been treated with oral levodopa–entacapone–carbidopa before LECIG initiation. Other PD‐related and non‐PD‐related variables are shown in Table 1.

**TABLE 1:** Data about sociodemographic aspects, comorbidities, PD, antiparkinsonian drugs, and other therapies at baseline (V0).

Characteristic	*n*	Value	Characteristic	*n*	Value
Age, years	73	70.1 ± 9.1 (42–85)	Time with fluctuations, years	69	7.2 ± 4 (2–20)
Gender, males, %	73	61.6	Nonmotor fluctuations, %	73	72.6
			Daily OFF time, h	70	5.3 ± 2.9 (1–15)
Weight, kg	51	68.2 ± 12.3	H&Y‐OFF	73	3 [3–4]
Height, cm	52	165.1 ± 9.3 (142–185)	H&Y‐ON	73	2 [2–3]
BMI	50	25.2 ± 3.9 (18.9–36)	UPDRS‐III‐OFF	60	42.5 ± 16.4
			UPDRS‐III‐ON	62	20.7 ± 11.1
Civil status, %	64		Dyskinesia, %	66	83.6
Married		65.6			
Single		15.6	Entacapone previously, %	70	63
Widowed		9.4	DAT previously, %	73	
Other		9.4	DBS		4.1
			Apomorphine		6.8
Living style, %	69		LCIG		21.9
With partner		60.9	More than 1		13.7
Alone		10.1	Other		1.4
With another family member		8.7			
Other		20.3	Treatment for PD, %	73	
			Levodopa		100
Comorbidities, %	73	26	LCIG		35.6
Arterial hypertension		15.1	MAO‐B inhibitor		60.8
Diabetes mellitus		25.7	Dopamine agonist		52.9
Dyslipidemia		8.2	COMT inhibitor		54.9
Atrial fibrillation		8.2	Entacapone		29.4
Cardiopathy		2.7	Opicapone		25.5
Lung disease		8.7	Amantadine		23.5
Polyneuropathy					
			L‐dopa daily dose, mg	66	1077.7 ± 454.2
Time from diagnosis, years	73	14.4 ± 6.3 (5–31)	DA daily dose, mg	39	237.7 ± 209.9
Motor phenotype, %	72		LEDD, mg	63	1492.6 ± 489.2
Tremor dominant		27.8			
Indeterminate		33.3	Other treatments, %	73	
PIGD		38.9	Antidepressant		54.8
Cognitive impairment, %	73		Benzodiazepine		47.9
MCI, %		31.5	Antipsychotic		24.7
Dementia, %		1.4	Antidementia		15.1

*Note*: The results represent %, mean ± SD (range), or median [interquartile range].

Abbreviations: BMI, body mass index; COMT, catechol‐O‐methyl transferase; DA, dopamine agonist; DAT, device‐aided therapy; DBS, deep brain stimulation; H&Y, Hoehn&Yahr; LCIG, levodopa–carbidopa infusion gel; LEDD, levodopa equivalent daily dose; MAO‐B, monoamine oxidase type B; MCI, mild cognitive impairment; PD, Parkinson disease; PIGD, postural instability gait difficulty; UPDRS‐III, Unified Parkinson's Disease Rating Scale Part III.

Regarding the initiation of LECIG, it was a direct initiation in 47 patients (64.4%) and a switch from LCIG in 26 patients (35.6%). The main cause of switching from LCIG to LECIG was: 38.5% (*n* = 10) nonoptimal control of OFF episodes, 30.8% (*n* = 8) characteristics of the pump, 11.5% (*n* = 3) biphasic dyskinesia, 11.5% (*n* = 3) other complications (freezing of gait, *n* = 1; polyneuropathy, *n* = 1; dopaminergic dysregulation syndrome, *n* = 1), and 7.7% (*n* = 2) unknown. The initiation of LECIG was with hospitalization in 40 patients (54.8%) and on an outpatient basis in 33 patients (45.2%). By groups, hospitalization was 68.1% (32/47) in patients with direct initiation of LECIG and in contrast 69.2% (18/26) on an outpatient basis in those patients switching from LCIG to LECIG. The mean time from indication to initiation the therapy was 50.8 ± 52.5 days (range = 1–365), whereas for full LECIG optimization it was 5.3 ± 5.8 days (range = 1–30). At V1, monotherapy with LECIG was reported in 26.4% of the patients and 24‐h infusion in only 4.2% (Table 2). Other data about LECIG initiation are shown in Table 3.

**TABLE 2:** Data on the initiation of LECIG at V1.

Factor	*n*	Value
Days from indication to therapy	73	50.8 ± 52.5 (1–365)
Days for full LECIG optimization	73	5.3 ± 5.8 (1–30)
Morning dose, mL	73	10.8 ± 3.7 (2.1–18)
Infusion rate, mL/h	72	2.3 ± 0.7 (0.6–4.25)
Extra dose, mL	72	1.5 ± 0.7 (0–3.5)
>1 flow, %	73	26
LECIG monotherapy, %	72	26.4
Levodopa + COMT inhibitor alone (%)	72	52.8
LECIG 24 h, %	72	4.2
Other treatments		
Levodopa retarded	72	50
MAO‐B inhibitor	68	20.6
Dopamine agonist	72	29.2
Amantadine	72	18.1
LEDD only considering LECIG, mg	65	1198.1 ± 330.4
LEDD, mg	65	1393.3 ± 414.9

*Note*: The results represent % or mean ± SD (range).

Abbreviations: COMT, catechol‐O‐methyl transferase; LECIG, levodopa–entacapone–carbidopa intestinal gel; LEDD, levodopa equivalent daily dose; MAO‐B, monoamine oxidase type B.

**TABLE 3:** AEs collected by the neurologist in patients receiving LECIG from V1 (initiation LECIG) to V2 (follow‐up visit; 177.3 ± 110.5 days, range = 7–476).

AE	*n* (%)
Total AEs	54
Tube migration	7
Significant weight loss	6
Tube obstruction	5
Erythema	5
Dyskinesia impairment	5
Stoma infection	4
Orthostatic hypotension/hypotension	4
Visual hallucinations/psychosis	4
Granuloma	3
Constipation	2
Vitamin B deficiency	2
Apathy	1
Paresthesias in lower extremities	1
Acute renal insufficiency	1
Acute urinary retention with urological complication	1
Acute ischemic stroke	1
Ischemic colitis	1
Pneumoperitoneum	1
Patients with at least one AE	36 (49.3)
At least possibly related [LECIG and/or device] AEs	52
Patients with at least possibly related [LECIG and/or device] AEs	34 (46.6)
Patients with at least one AE leading to discontinuation	5 (6.8)
Serious stoma infection	1
Acute urinary retention with urological complication	1
Psychosis	1
Dyskinesia	1
Ischemic colitis	1
Patients with at least one possibly related [LECIG and/or device] leading to discontinuation	3 (4.1)
Deaths	1 (1.4)^a^

Abbreviations: AE, adverse event; LECIG, levodopa–‐entacapone–carbidopa intestinal gel.

^a^ The patient had a severe stoma infection that required removing the percutaneous endoscopic gastrostomy and stopping LECIG. He was institutionalized and died >1 month later related to acute renal failure.

**FIGURE 1:**
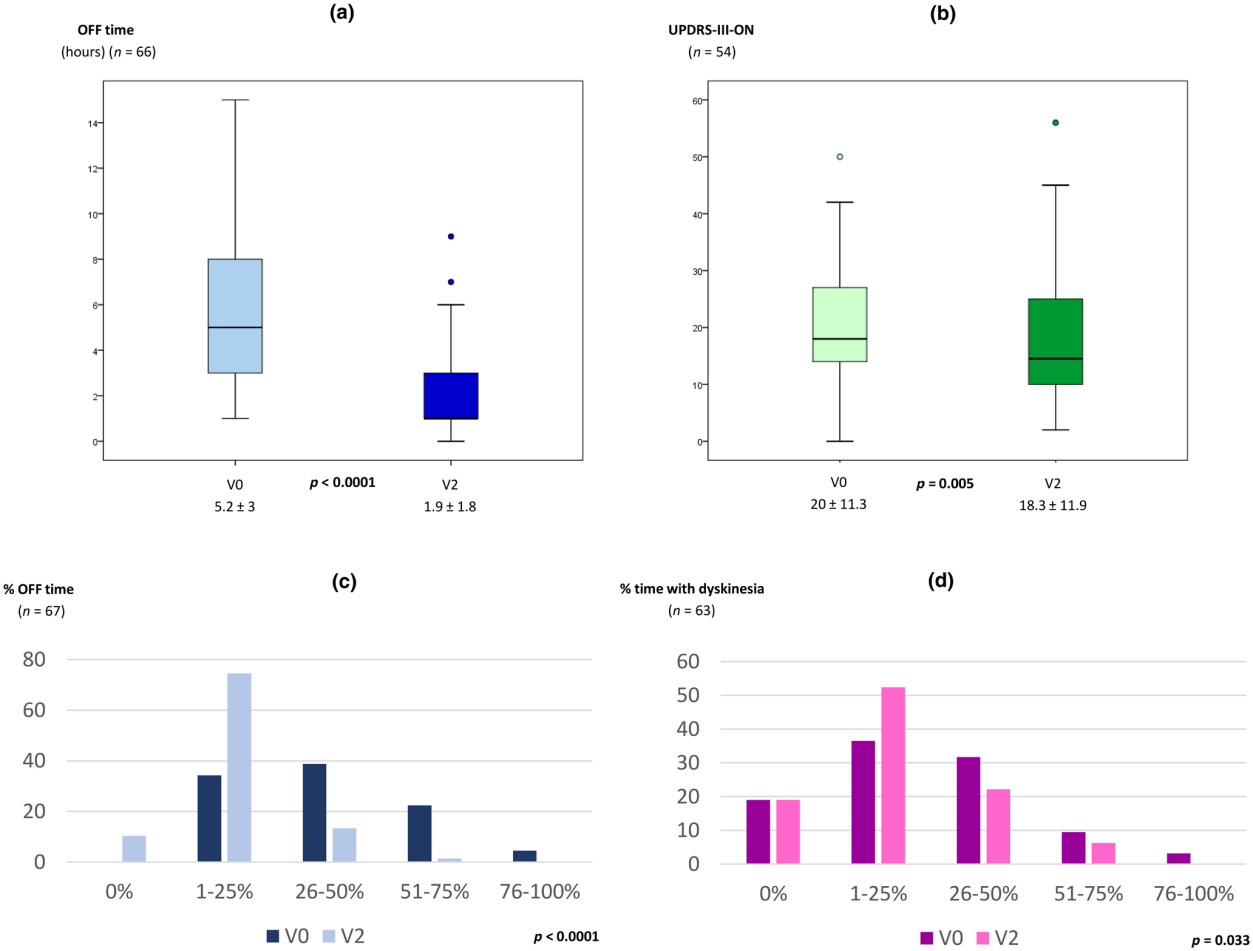
(a) Change in the mean OFF time from V0 to V2 (*n* = 66, *p* < 0.0001). (b) Change in the mean Unified Parkinson's Disease Rating Scale Part III conducted during the ON state (UPDRS‐III‐ON) from V0 to V2 (*n* = 54, *p* = 0.005). (c) Change in the frequency of percentage of the day in the OFF state from V0 to V2 (*n* = 67, *p* < 0.0001). (d) Change in the frequency of percentage of the day with dyskinesia from V0 to V2 (*n* = 63, *p* = 0.033). Data are presented in panels a and b as boxplots, with the box representing the median and the two middle quartiles (25%–75%). Probability values were computed using the Wilcoxon signed‐rank test (a and b) and the marginal homogeneity test (c and d). Mild outliers (circles) are data points that are more extreme than Q1–1.5.

**FIGURE 2:**
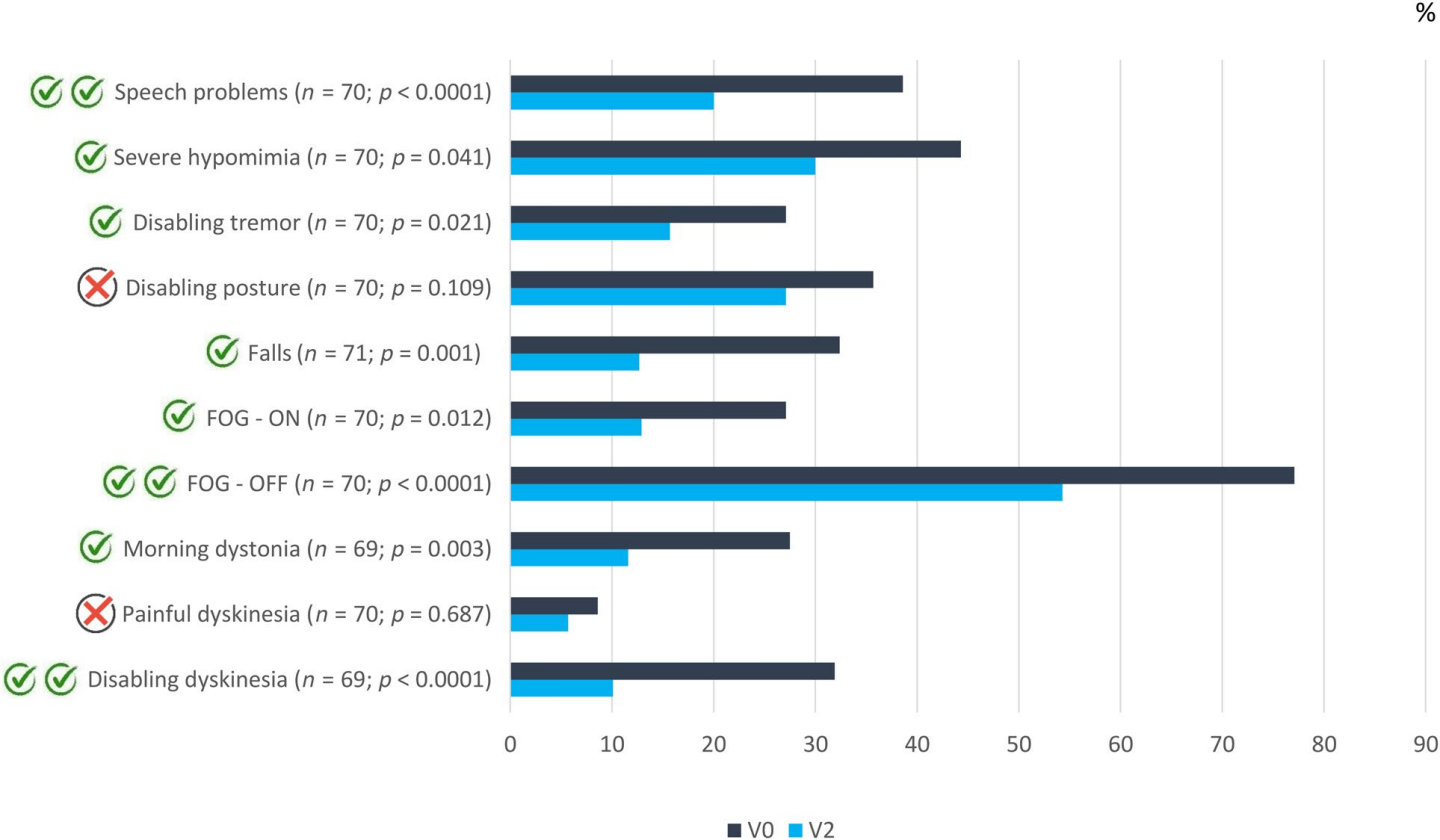
Percentage of patients suffering from different disabling motor symptoms at V2 versus V0. Red symbols, *p* ≥ 0.05. Green symbols, *p* < 0.05 (two symbols, *p* < 0.0001). The marginal homogeneity test was applied. FOG–OFF, freezing of gait during the OFF state; FOG–ON, freezing of gait during the ON state.

**FIGURE 3:**
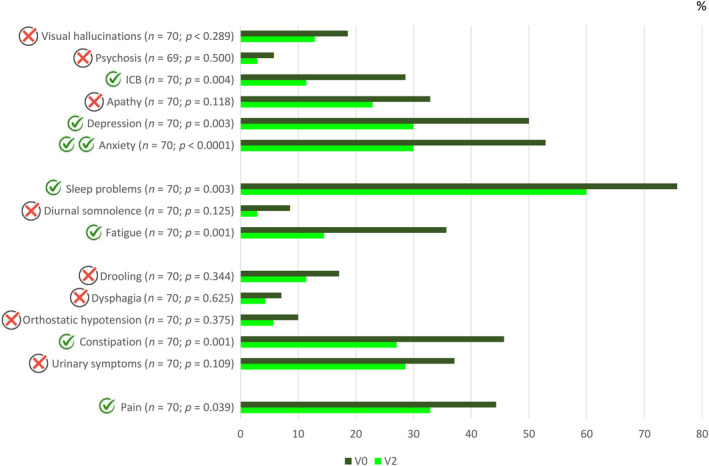
Percentage of patients suffering from different disabling nonmotor symptoms at V2 versus V0. Red symbols, *p* ≥ 0.05. Green symbols, *p* < 0.05 (two symbols, *p* < 0.0001). The marginal homogeneity test was applied. ICB, impulse control behavior.

**FIGURE 4:**
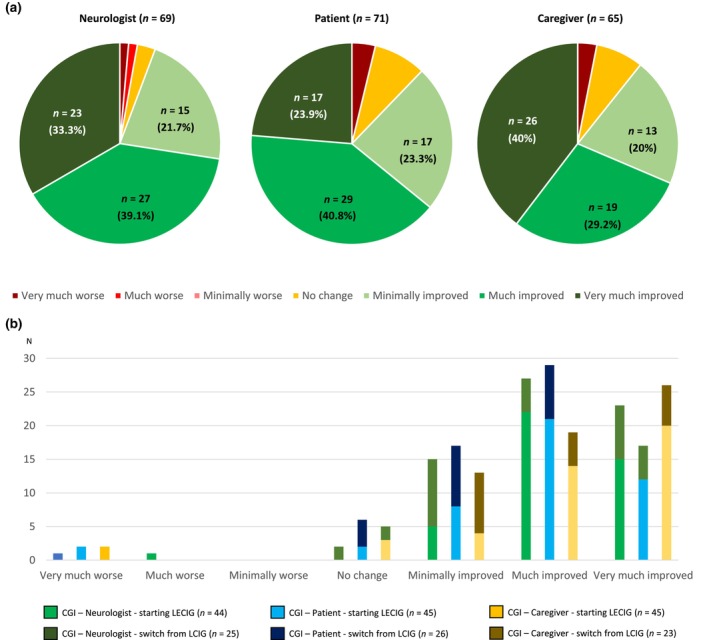
(a) Clinical Global Impression of Change (CGI) according to the opinion of the neurologist (*n* = 69), patient (*n* = 71), and caregiver (*n* = 65) at V2 (follow‐up visit). (b) Patients with a direct initiation of levodopa–entacapone–carbidopa intestinal gel (LECIG) appear in light colors, and those who switched from levodopa–carbidopa intestinal gel (LCIG) to LECIG appear in dark colors. Improvement was observed in 94.1%, 88%, and 89.2% of patients according to the opinion collected by the neurologist, own patient and principal caregiver, respectively.

**FIGURE 5:**
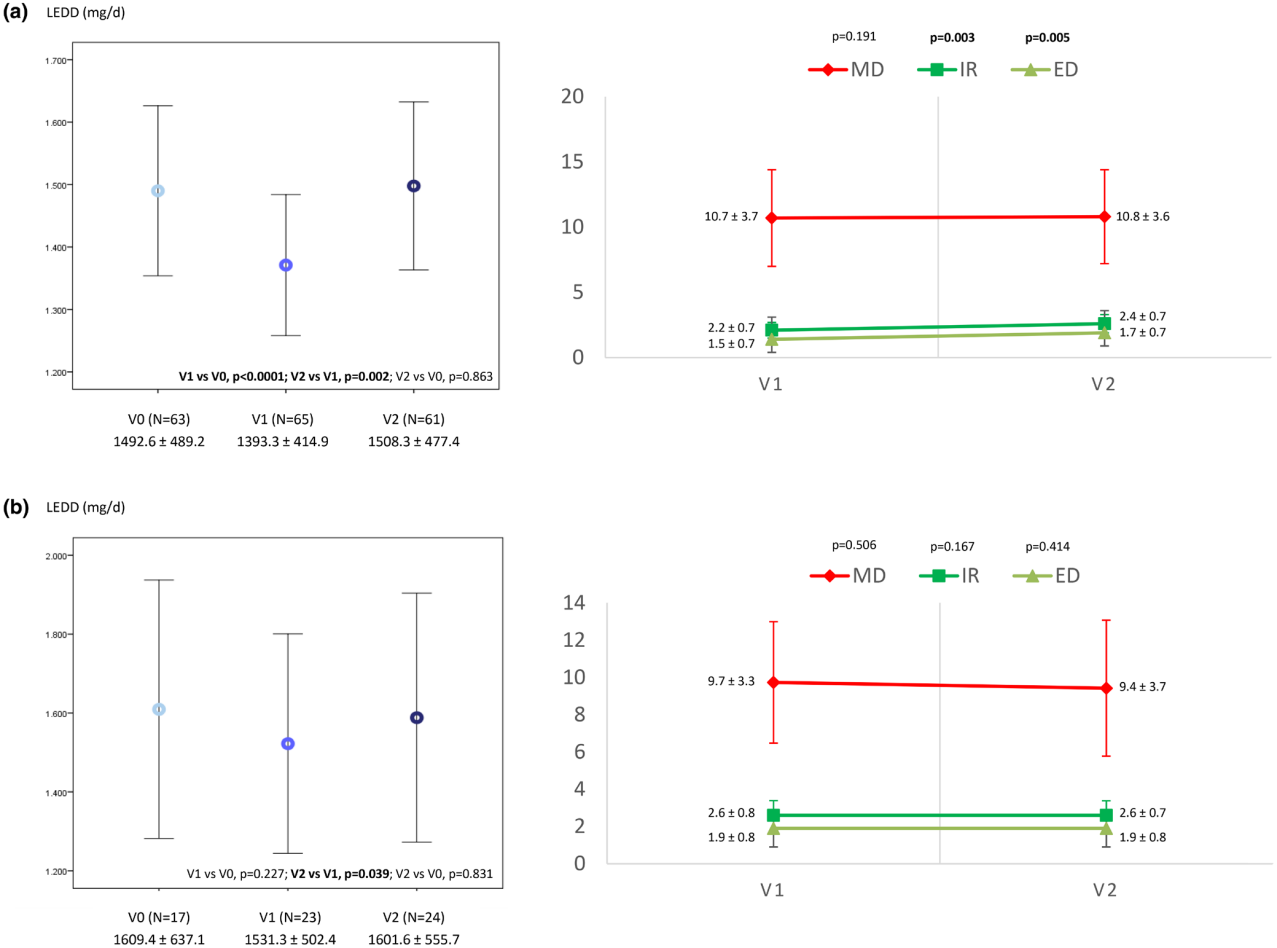
Levodopa equivalent daily dose (LEDD) at V0, V1, and V2 and morning dose (mL), infusion rate (mL/h), and extra dose at V1 and at V2 in the whole cohort (a) and the subgroup of patients switching from levodopa–carbidopa intestinal gel to levodopa–entacapone–carbidopa intestinal gel (b). ED, extra dose; IR, infusion rate; MD, morning dose.

The mean exposure to LECIG was 177.3 ± 110.5 days (range = 7–476). Follow‐up time was ≥3 and ≥6 months in 76.1% and 47.8% of the patients, respectively. From V0 to V2, a significant reduction in OFF time was observed (5.2 ± 3 vs. 1.9 ± 1.8 h, *n* = 66, *p* < 0.0001; Figure 1a). A decrease in the percentage of the day awake in the OFF state (*n* = 67, *p* < 0.0001; Figure 1c) and with dyskinesia (*n* = 63, *p* = 0.033; Figure 1d) was also observed. The mean score in the UPDRS‐III during the ON state was also lower at V2 than at V0 (18.3 ± 11.9 vs. 20 ± 11.3, *n* = 54, *p* = 0.005; Figure 1b). OFF time and the UPDRS‐III score were lower at V2 compared to before LECIG initiation in both groups, patients with direct initiation of LECIG (Figure S1) and those switching from V0 to V2 (Figure S2), but no differences were detected regarding time with dyskinesia. The frequency of patients suffering from different disabling motor symptoms (Figure 2) and NMSs (Figure 3) decreased from V0 to V2. More than 70% of the patients presented from “much improvement” to “very much improvement” based on the CGI‐C according to the opinion of the neurologist, caregiver, and patient (Figure 4). Although no significant change was detected in the LEDD from V0 to V2, an increase in the infusion rate (*p* = 0.003) and extra dose (*p* = 0.005) from V1 to V2 was observed (Figure 5). Specifically, the dose of levodopa (mg/day) administered by infusion in the group of patients who switched from LCIG to LECIG decreased from 1329.9 ± 517.7 at V0 to 921.3 ± 343.9 at V1 (30.7% reduction, *p* = 0.001). The frequency of patients receiving a monoamine oxidase type B inhibitor and a dopamine agonist decreased from 60% to 23% (*p* < 0.0001) and from 56% to 25% (*p* < 0.0001), respectively. At V2, more than one cartridge was used by 24.7% of the patients and 7% were on 24‐h infusion therapy. In the group of patients who switched from LECIG to LCIG, all patients reported being satisfied with the pump: 13.6% minimally better, 36.6% much better, and 50% very much better.

A total of 54 AEs were reported in 36 patients (49.3% of the sample; Table 3). The frequency of patients with at least one AE related to LECIG and/or dispositive was 46.6% (36/73). Twenty patients (27.4%), had at least one LECIG/dispositive‐related systemic AE, whereas 18 patients (24.7%) had at least one LECIG/dispositive‐related local AE. Frequency of all AEs was <10% (Figure S3). Five patients (6.8%) discontinued LECIG, three (4.1%) by a LECIG/dispositive‐related AE. One patient died in the context of an acute hospitalization due to a severe stoma infection and acute renal failure. Although significant weight loss was reported in six patients, mean weight did not change overtime (67.6 ± 11.9 at V0 vs. 66.1 ± 11.6 at V1, *n* = 42, *p* = 0.093). Finally, a significant change was detected from V0 to V2 in the plasma levels of homocysteine (12.8 ± 4.0 mmol/L at V2 vs. 14.9 ± 4.6 mmol/L at V0, *n* = 15, *p* = 0.048) but not in the plasma levels of B1 (25.4 ± 17.6 ng/mL at V2 vs. 32.2 ± 21.3 ng/mL at V0, *n* = 7, *p* = 0.735), B6 (26.3 ± 29.2 ng/mL at V2 vs. 20.8 ± 20.1 ng/mL at V0, *n* = 14, *p* = 0.807), and B12 vitamins (384.6 ± 193.1 pg/mL at V2 vs. 389 ± 229.7 pg/mL at V0, *n* = 27, *p* = 0.899).

## DISCUSSION

In the present study, we observed that LECIG infusion therapy was effective, safe, and well tolerated in advanced PD patients managed under real clinical practice. The frequency of many disabling motor symptoms and NMSs decreased under LECIG treatment, and interestingly, LECIG reduced OFF time not only in the group of patients with a direct initiation but also in those patients who switched from LCIG to LECIG. Despite the methodological limitations, this is the largest RWE study conducted so far on the effect of LECIG in PD patients in which also much information was collected.

The characteristics of the patients treated with LECIG were quite similar to advanced PD patients treated with subcutaneous apomorphine and/or LCIG in daily clinical practice, with a mean disease duration of approximately 14 years and >5 h during the OFF state per day [18, 19, 20]. At baseline, patients had a good response to levodopa according to the change in the H&Y stage and UPDRS‐III score from the OFF to the ON state, a critical factor regarding a proper indication for a DAT. The use of other anti‐PD medications and other drugs was frequent as in other PD cohorts treated with LCIG [21] and LECIG [11, 12, 13, 15]. Moreover, >40% of the patients had been treated with a previous DAT, the most frequent being LCIG. As in previous studies of LECIG [11, 13], in our cohort a high percentage (up to 35.6%) of patients switched from LCIG to LECIG. These results suggest that the new DAT LECIG could be an alternative therapy to other DATs for advanced PD, but more time is needed to determine whether specific criteria could be applied to select one enteral therapy over the other (i.e., LECIG vs. LCIG).

There is a lack of evidence about the effect of LECIG on advanced PD patients. To our knowledge, only eight studies about LECIG have been published [8, 9, 10, 11, 12, 13, 14, 15]. Of them, four were about real clinical experience [11, 12, 13, 15], two were pharmacokinetic studies [9, 10], one was designed to compare two types of extension tubes in PD patients treated with LECIG [14], and the other one was a review [8]. Our sample (*N* = 73) is the largest until now after Szász et al., with 74 patients treated [12]. However, Szász et al. only collected data before and immediately after starting with LECIG, not on the follow‐up. Very importantly, a significant reduction was observed in our study in OFF time per day from 5.2 to 1.9 h (3.3 h) after a mean follow‐up of nearly 6 months. The change in daily OFF time has been reported in only two other studies of LECIG, with reductions of 4 h (from 5.7 to 1.7 h, *N* = 74) [12] and 2.1 h (from 3.3 to 1.2 h, *N* = 20) [15] per day. The UPDRS‐III‐ON score decreased also after LECIG in patients from our cohort, an aspect not previously reported with LECIG but observed with LCIG in PD patients compared to oral levodopa [22]. Moreover, we also detected a significant reduction in the percentage of the day with dyskinesia and in the frequency of many motor symptoms and NMSs at the final visit (post‐LECIG) compared to the baseline status (pre‐LECIG). Again, this aspect has not been properly collected in other studies published on LECIG. Furthermore, this benefit was accompanied by the perception of improvement by the majority of patients, caregivers, and neurologists according to the CGI‐C.

An interesting aspect is the improvement observed in the subgroup of patients previously treated with LCIG who switched to LECIG (*n* = 26). Again, this is the largest group of patients reported to switch from LCIG to LECIG (26/73), with the sample being *n* = 12 [11], *n* = 6 [13], and *n* = 0 [12, 15] in previous RWE studies. Importantly, LECIG was indicated in more than half of the patients due to complications and/or lack of proper control of symptoms. Despite this, a significant reduction in OFF time and improvement according to the CGI‐C was found at V2 in most patients. This benefit was obtained without a significant increase in the LEDD from the baseline to V2. Previous data suggest that the continuous maintenance dose of levodopa should be reduced by approximately 35%, on a population level, when entacapone is simultaneously infused [9] compared to the previously suggested reduction of 20% when an LCIG infusion is administered with oral entacapone [23]. In our cohort, the reduction in levodopa dose was 31%. A recently developed model investigating entacapone pharmacokinetics suggested that 6%–11% is lost due to intestinal metabolism [24]. As Senek et al. suggested [9], the immediate delivery of entacapone to the small intestine with the infusion, and perhaps a shorter intestinal residence time, may result in a higher bioavailability of entacapone, and thereby higher inhibition of COMT compared with oral administration. This could explain a stronger effect of LECIG compared to LCIG for the theorical LEDD calculated [17]. Interestingly, the three patients with biphasic dyskinesia who switched from LCIG to LECIG improved according to the CGI‐C (minimally improved, *n* = 1; much improved, *n* = 1; very much improved, *n* = 1). In any case, more experience worldwide and data from other ongoing studies such as the ELEGANCE study [16] will help us to determine the effect of LECIG compared to LCIG. On the other hand, more than 1 in 3 patients switched from LCIG to LECIG due to the pump (i.e., size, weight, only manage one flow, etc.) and all patients showed better satisfaction with the new pump to administer LECIG. As a whole, our data suggest that LECIG could be at least an alternative DAT in patients previously treated with LCIG or other DATs without optimal control.

With regard to safety and tolerability, our study demonstrated a good security profile for LECIG. Fewer than 7% of the patients dropped out of therapy after a mean follow‐up of approximately 6 months, clearly lower that 33% (10/30) reported in a study conducted in Finland [13] and 25% (6/24) in another one in Sweden [11]. Although a longer follow‐up is required, the extensive previous experience of neurology teams from Spain in the management of PD patients with LCIG could be a key factor to obtain this result. Surprisingly, no AEs were reported in two studies conducted in Romania [11, 15], although they only collected information about the initiation of the therapy (i.e., the final evaluation was performed the day before discharge). Otherwise, safety issues encountered were similar to those reported with LECIG in previous studies [8, 11, 13]. In our cohort, approximately 1 in 4 patients had a local and a systemic AE related to LECIG and/or the device system, so from a clinical point of view, it is necessary to be alert to possible complications in advanced PD patients treated with LECIG. It is noteworthy that although up to 1 in 3 patients treated with LECIG had not previously received entacapone, no cases of diarrhea were detected. Three of 24 patients from the Öthman et al. cohort discontinued LECIG due to diarrhea (half of the discontinuations) [11]. On the other hand, no cases were detected by Viljaharju et al. (*N* = 30) [13]. At some centers of our study, a tolerability test with oral entacapone was performed before starting LECIG, which could help to determine the potential tolerability of the therapy.

This study has some important limitations. The first are limitations related to the retrospective observational design. However, much information was available and collected from the medical records due to patients being exhaustively evaluated at centers in Spain with experience in the management of DATs. Second, although this is the second largest study conducted until now of PD patients treated with LECIG, the sample for some analyses was low (e.g., subgroups of patients regarding the type of initiation). Third, nonparametric tests were applied to analyze the change in different variables from V0 to V2 according to the observational design, so the influence of covariates was not taken into consideration. Fourth, no conclusions can be drawn about vitamin plasma markers, because the samples were small, and some patients were already receiving treatment with vitamin supplements before starting LECIG. However, it would be interesting in relation to our findings to analyze long‐term changes in homocysteine plasma levels and complications (i.e., cognitive impairment, polyneuropathy, etc.) in patients treated with LECIG compared to LCIG in a large cohort. Finally, mean time on LECIG was approximately 6 months, so a longer follow‐up period should be necessary to determine the response of patients to LECIG in the long term. In contrast, this study reports so far the most complete RWE on the effect of LECIG on advanced PD patients.

In conclusion, in the present study we observed that LECIG infusion therapy was effective, safe, and well tolerated in advanced PD patients managed under real clinical practice. More data are needed to determine the effect of LECIG on PD patients in the long term.

## AUTHOR CONTRIBUTIONS

**Diego Santos‐García:** Conceptualization; investigation; writing – original draft; methodology; validation; visualization; writing – review and editing; software; formal analysis; project administration; data curation; supervision; resources. **Lydia López‐Manzanares:** Investigation; writing – review and editing; methodology. **Inés Muro:** Investigation; writing – review and editing; methodology. **Pablo Lorenzo‐Barreto:** Investigation; methodology; writing – review and editing. **Elena Casas Peña:** Investigation; methodology; writing – review and editing. **Rocío García‐Ramos:** Investigation; writing – review and editing; methodology. **Tamara Fernández Valle:** Investigation; methodology; writing – review and editing. **Carlos Morata‐Martínez:** Methodology; investigation; writing – review and editing. **Raquel Baviera‐Muñoz:** Investigation; methodology; writing – review and editing. **Irene Martínez‐Torres:** Investigation; methodology; writing – review and editing. **María Álvarez‐Sauco:** Methodology; writing – review and editing; investigation. **Déborah Alonso‐Modino:** Investigation; methodology; writing – review and editing. **Inés Legarda:** Methodology; writing – review and editing; investigation. **María Fuensanta Valero‐García:** Investigation; writing – review and editing; methodology. **José Andrés Suárez‐Muñoz:** Methodology; investigation. **Juan Carlos Martínez‐Castrillo:** Methodology; investigation; writing – review and editing. **Ana Belén Perona:** Methodology; investigation; writing – review and editing. **Jose María Salom:** Methodology; writing – review and editing; investigation. **Esther Cubo:** Investigation; writing – review and editing; methodology. **Caridad Valero‐Merino:** Methodology; investigation; writing – review and editing. **Nuria López‐Ariztegui:** Methodology; investigation; writing – review and editing. **Pilar Sánchez Alonso:** Methodology; writing – review and editing; investigation. **Sabela Novo Ponte:** Investigation; writing – review and editing; methodology. **Elisa Gamo González:** Investigation; methodology; writing – review and editing. **Raquel Martín García:** Methodology; investigation; writing – review and editing. **Raúl Espinosa:** Methodology; writing – review and editing; investigation. **Mar Carmona:** Methodology; writing – review and editing; investigation. **Cici Esmerali Feliz:** Investigation; methodology; writing – review and editing. **Pedro García Ruíz:** Methodology; writing – review and editing; investigation. **Teresa Muñoz Ruíz:** Investigation; methodology; writing – review and editing. **Beatriz Fernández Rodríguez:** Investigation; methodology; writing – review and editing. **Marina Mata:** Investigation; methodology; writing – review and editing.

## CONFLICT OF INTEREST STATEMENT

D.S.‐G. has received honoraria for educational presentations and advisory services from AbbVie, UCB Pharma, Lundbeck, KRKA, Zambon, Bial, Italfarmaco, Teva, Archímedes, Esteve, Stada, and Merz, and grants from the Spanish Ministry of Economy and Competitiveness (PI16/01575) cofounded by Instituto de Salud Carlos III (Concesión de subvenciones de Proyectos de Investigación en Salud de la convocatoria 2020 de la Acción Estratégica en Salud 2017–2020 por el proyecto “Progresión No Motora e Impacto en la Calidad de Vida en la Enfermedad de Parkinson”; Concesión de Contrato para la intensificación de la actividad investigadora en el Sistema Nacional de Salud, Convocatoria 2021, Instituto de Salud Carlos III). L.L.‐M. has received compensation for advisory services or consulting, research grant support, or speaker honoraria from AbbVie, Acorda, Bial, Intec Pharma, Italfarmaco, Pfizer, Roche, Teva, UCB, and Zambon. I.M. has received honoraria for lectures with educational purposes and advisory services from AbbVie, Bial, Lundbeck, Zambon, and Stada. P.L.‐B. has received honoraria for educational presentations and advisory services from Zambon and Orion Pharma. E.C.P. has received honoraria for educational presentations and advisory services from Bial. R.G.‐R. has received honoraria and grants for lecturing and advisory services from AbbVie, Zambón, Bial, Merk, and Stada. R.B.‐M. is supported by a Rio Hortega contract (CM22/00099) from Instituto de Salud Carlos III. I.M.‐T. has received honoraria from AbbVie, Bial, Ipsen, Medtronic, Merz, and Pallex for lecturing. M.Á.‐S. has received honoraria for educational presentations and advisory services from AbbVie, UCB Pharma, Zambon, Bial, and Teva. I.L. has received honoraria for educational presentations and advisory services from AbbVie, UCB Pharma, Zambon, Bial, and Teva. M.F.V.‐G. has received honoraria for educational presentations from Bial. J.C.M.‐C. has received research support from Lundbeck, Italfarmaco, Allergan, Zambon, Merz, and AbbVie. He has received speaking honoraria from AbbVie, Bial, Italfarmaco, Lundbeck, Krka, Teva, UCB, Zambon, Allergan, Ipsen, and Merz. A.B.P. has received honoraria from AbbVie, Bial, Stada, and Merz for lecturing. J.M.S. has received speaking and/or advisory honoraria from Italfarmaco, Bial, Zambon, AbbVie, Esteve, Stada, and Italfarmaco. E.C. has received travel grants from AbbVie, Allergan, and Boston Pharmaceuticals and lecturing honoraria from AbbVie and International Parkinson's Disease Movement Disorder Society. C.V.‐M. has received honoraria for educational services from Zambon, AbbVie, and UCB. N.L.‐A. has received compensation for advisory services or consulting, research grant support, or speaker honoraria from AbbVie, Italfarmaco, Stada, Bial, Zambon, UCB, and Lundbeck. P.S.A. has received honoraria for educational presentations and advisory services from AbbVie, UCB Pharma, Lundbeck, Krka, Zambon, Bial, and Teva. S.N.P. has received honoraria for educational presentations from AbbVie, Alter, and Schwabe Pharma Iberica and travel grants from Bial, Zambón, and Esteve. E.G.G. has received honoraria for educational presentations and advisory services by Italfarmaco, Bial, and Health in Code. R.M.G. has received honoraria for educational presentations and advisory services from Almirall. R.E. has received honoraria for educational presentations or advisory services or travel grants from AbbVie, Stada, Orion, Merz, Bial, Italfarmaco, Novartis, Pfizer, and Lundbeck. M.C. has received honoraria from AbbVie, Bial, Esteve, and Italfarmaco for lecturing and from Bial and Stada for consultant/scientific advisory services. C.E.F. has received honoraria for educational presentations from AbbVie, Bial, Steve, Zambom, and Alter. P.G.R. has received personal compensation for consultancy/scientific advisory board participation from Italfarmaco, Britannia, Bial, Stada, and Esteve and speaking honoraria from Italfarmaco, Bial, Stada, and Esteve. M.M. has received honoraria for educational presentations or advisory services from Stada, AbbVie, Orion Pharma, Italfarmaco, Bial, Zambon, and Esteve. None of the other authors has any conflict of interest to disclose.

## Supporting information


**FIGURE S1.** Change from V0 to V2 in motor complications and motor status in the subgroup of patients with a direct initiation of levodopa–entacapone–carbidopa intestinal gel. (a) Change in the mean OFF time from V0 to V2 (*n* = 43, *p* < 0.0001). (b) Change in the mean UPDRS‐III‐ON from V0 to V2 (*n* = 34, *p* = 0.034). (c) Change in the frequency of percentage of the day in the OFF state from V0 to V2 (*n* = 43, *p* < 0.0001). (d) Change in the frequency of percentage of the day with dyskinesia from V0 to V2 (*n* = 41, *p* = 0.083). Data are presented in panels a and b as boxplots, with the box representing the median and the two middle quartiles (25%–75%). Probability values were computed using the Wilcoxon signed‐rank test (a and b) and the marginal homogeneity test (c and d). Mild outliers (circles) are data points that are more extreme than Q1–1.5. UPDRS‐III‐ON, Unified Parkinson’s Disease Rating Scale Part III conducted during the ON state.

**FIGURE S2.** Change from V0 to V2 in motor complications and motor status in the subgroup of patients switching from levodopa–carbidopa intestinal gel to levodopa–entacapone–carbidopa intestinal gel. (a) Change in the mean OFF time from V0 to V2 (*n* = 24, *p* < 0.0001). (b) Change in the mean UPDRS‐III‐ON from V0 to V2 (*n* = 20, *p* = 0.043). (c) Change in the frequency of percentage of the day in the OFF state from V0 to V2 (*n* = 24, *p* = 0.003). (d) Change in the frequency of percentage of the day with dyskinesia from V0 to V2 (*n* = 17, *p* = 0.194). Data are presented in panels a and b as boxplots, with the box representing the median and the two middle quartiles (25%–75%). Probability values were computed using the Wilcoxon signed‐rank test (a and b) and the marginal homogeneity test (c and d). Mild outliers (circles) are data points that are more extreme than Q1–1.5. UPDRS‐III‐ON, Unified Parkinson’s Disease Rating Scale Part III conducted during the ON state.

**FIGURE S3.** Frequency of local and systemic adverse events related to levodopa–entacapone–carbidopa intestinal gel (treatment and/or device) reported from V1 to V2.

## Data Availability

The protocol and the statistical analysis plan are available on request. Deidentified participant data are not available for legal and ethical reasons.
