# Supplementary figures and images for: Effectiveness and safety of levodopa–entacapone–carbidopa infusion in Parkinson disease: A real‐world data study
Source: Eur J Neurol. 2024 Oct 28;32(1):e16535. doi: 10.1111/ene.16535 (PMC11625960; doi:10.1111/ene.16535)

**Figure 1 - SM**

**A**

**OFF time**  
(hours) (N=43)

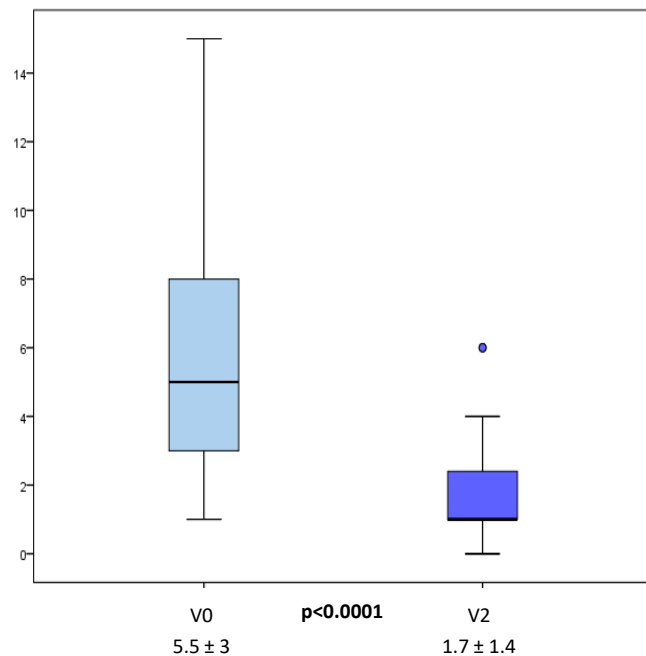

**B**

**UPDRS-III-ON**  
(N=34)

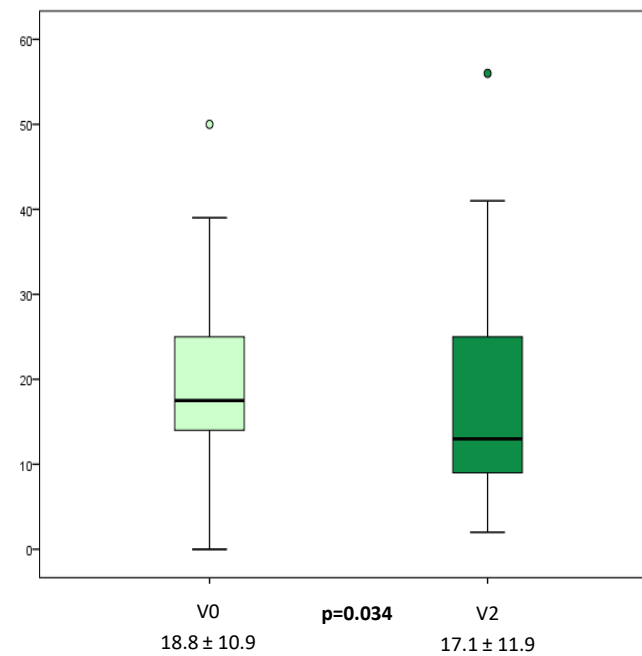

**C**

**% OFF time**  
(N=43)

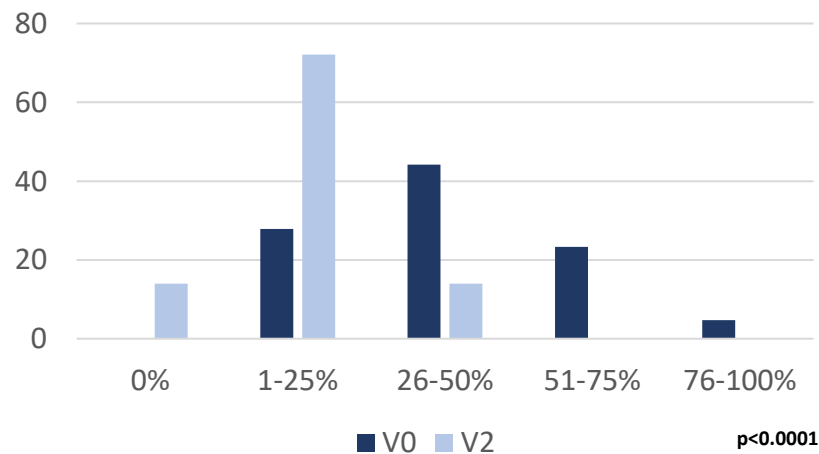

**D**

**% time with  
dyskinesia**  
(N=41)

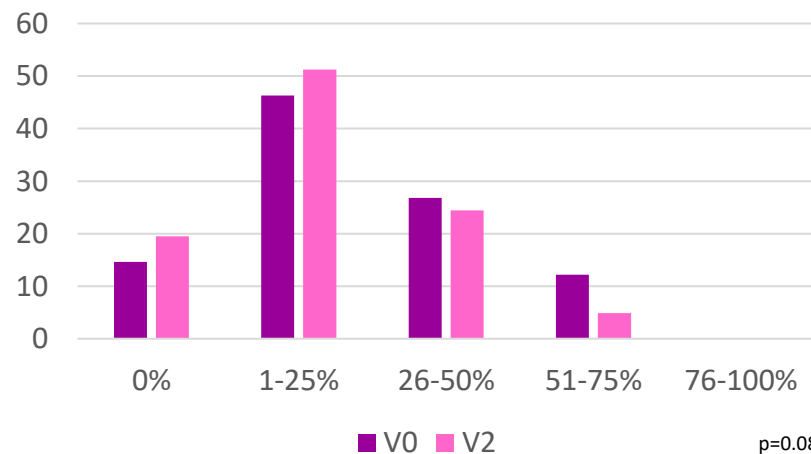

Supplement: Supplementary file 1 — FIGURE S1. Change from V0 to V2 in motor complications and motor status in the subgroup of patients with a direct initiation of levodopa–entacapone–carbidopa intestinal gel. (a) Change in the mean OFF time from V0 to V2 (n = 43, p < 0.0001). (b) Change in the mean UPDRS‐III‐ON from V0 to V2 (n = 34, p = 0.034). (c) Change in the frequency of percentage of the day in the OFF state from V0 to V2 (n = 43, p < 0.0001). (d) Change in the frequency of percentage of the day with dyskinesia from V0 to V2 (n = 41, p = 0.083). Data are presented in panels a and b as boxplots, with the box representing the median and the two middle quartiles (25%–75%). Probability values were computed using the Wilcoxon signed‐rank test (a and b) and the marginal homogeneity test (c and d). Mild outliers (circles) are data points that are more extreme than Q1–1.5. UPDRS‐III‐ON, Unified Parkinson’s Disease Rating Scale Part III conducted during the ON state. [file ENE-32-e16535-s003.pdf]

A

OFF time  
(hours) (N=24)

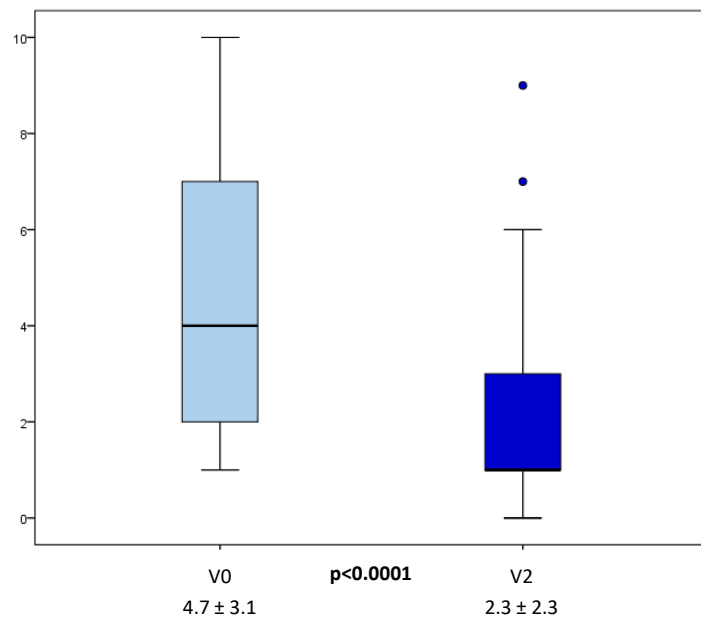

B

UPDRS-III-ON  
(N=20)

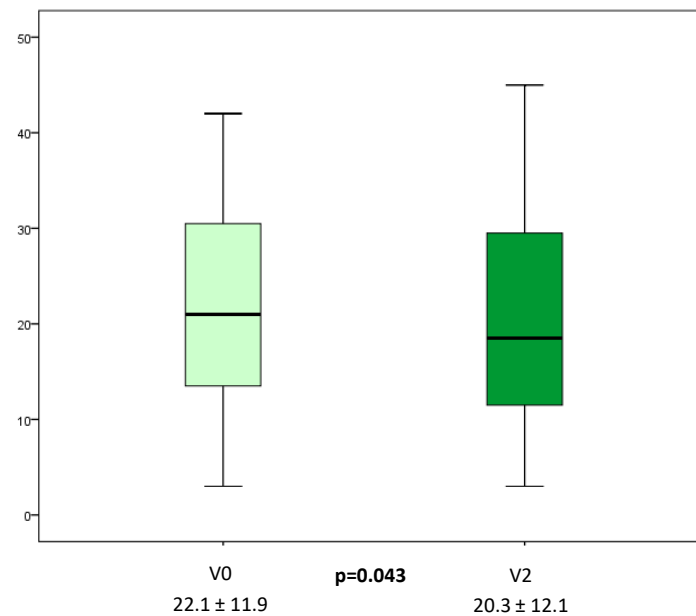

% OFF time  
(N=24)

C

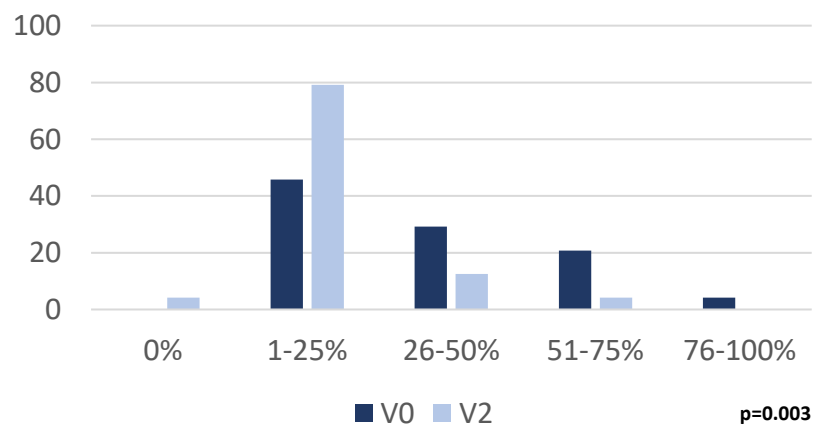

D

% time with LID  
(N=17)

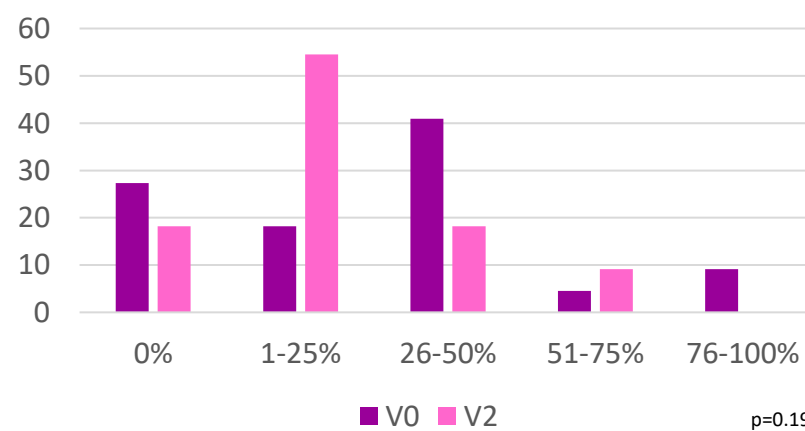

Supplement: Supplementary file 2 — FIGURE S2. Change from V0 to V2 in motor complications and motor status in the subgroup of patients switching from levodopa–carbidopa intestinal gel to levodopa–entacapone–carbidopa intestinal gel. (a) Change in the mean OFF time from V0 to V2 (n = 24, p < 0.0001). (b) Change in the mean UPDRS‐III‐ON from V0 to V2 (n = 20, p = 0.043). (c) Change in the frequency of percentage of the day in the OFF state from V0 to V2 (n = 24, p = 0.003). (d) Change in the frequency of percentage of the day with dyskinesia from V0 to V2 (n = 17, p = 0.194). Data are presented in panels a and b as boxplots, with the box representing the median and the two middle quartiles (25%–75%). Probability values were computed using the Wilcoxon signed‐rank test (a and b) and the marginal homogeneity test (c and d). Mild outliers (circles) are data points that are more extreme than Q1–1.5. UPDRS‐III‐ON, Unified Parkinson’s Disease Rating Scale Part III conducted during the ON state. [file ENE-32-e16535-s001.pdf]
