# Supplementary material for: Effectiveness and safety of levodopa–entacapone–carbidopa infusion in Parkinson disease: A real‐world data study
Source: Eur J Neurol. 2024 Oct 28;32(1):e16535. doi: 10.1111/ene.16535 (PMC11625960; doi:10.1111/ene.16535)
Supplement: Supplementary file 3 — FIGURE S3. Frequency of local and systemic adverse events related to levodopa–entacapone–carbidopa intestinal gel (treatment and/or device) reported from V1 to V2. [file ENE-32-e16535-s002.pdf]

Figure 3 – SM

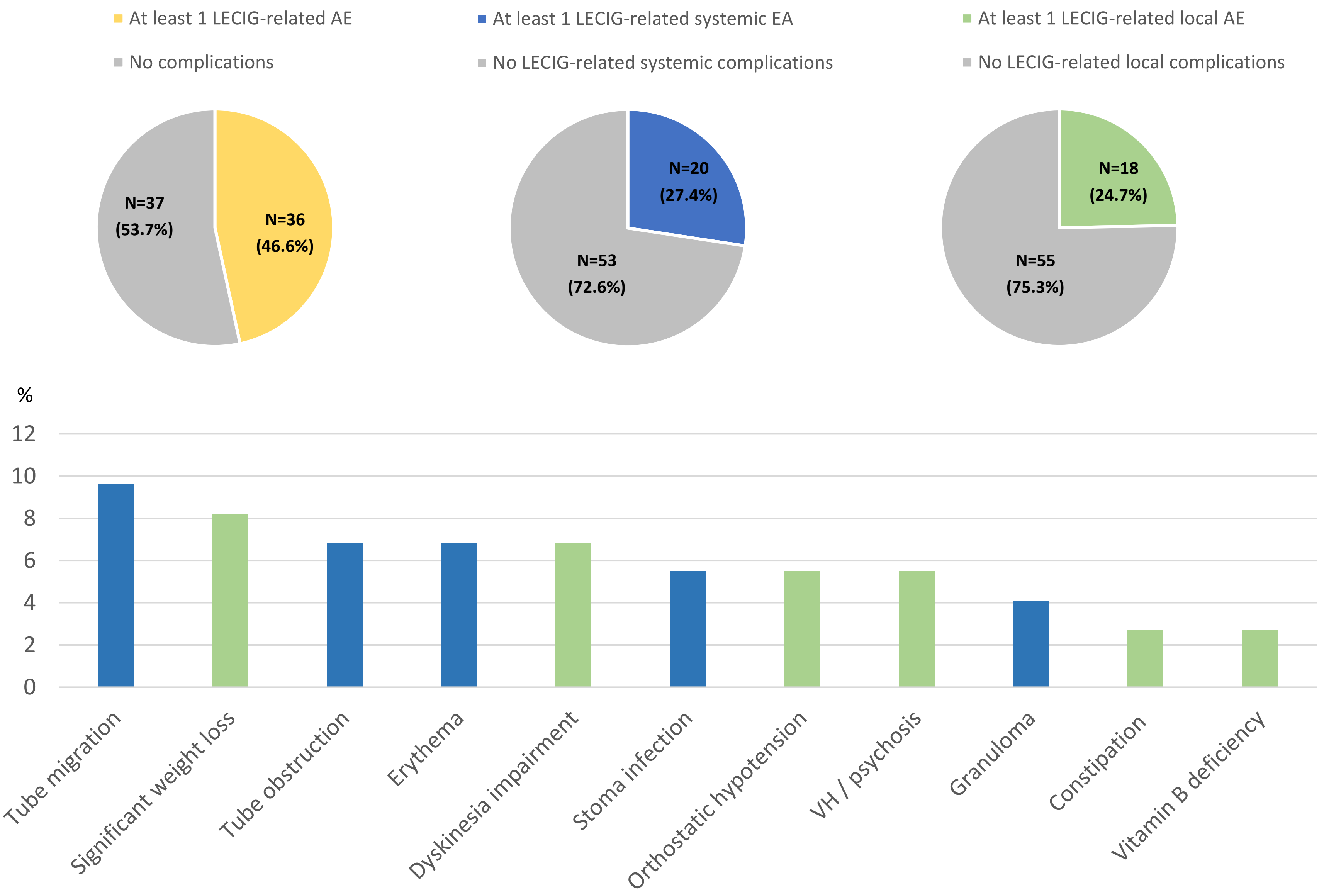

At least 1 LECIG-related AE
No complications
N=37
(53.7%)
N=36
(46.6%)
At least 1 LECIG-related systemic EA
No LECIG-related systemic complications
N=20
(27.4%)
N=53
(72.6%)
At least 1 LECIG-related local AE
No LECIG-related local complications
N=18
(24.7%)
N=55
(75.3%)
%
12
10
8
6
4
2
0
Tube migration
Significant weight loss
Tube obstruction
Erythema
Dyskinesia impairment
Stoma infection
Orthostatic hypotension
VH / psychosis
Granuloma
Constipation
Vitamin B deficiency
